# Genomic Prediction in Maize Breeding Populations with Genotyping-by-Sequencing

**DOI:** 10.1534/g3.113.008227

**Published:** 2013-11-01

**Authors:** José Crossa, Yoseph Beyene, Semagn Kassa, Paulino Pérez, John M. Hickey, Charles Chen, Gustavo de los Campos, Juan Burgueño, Vanessa S. Windhausen, Ed Buckler, Jean-Luc Jannink, Marco A. Lopez Cruz, Raman Babu

**Affiliations:** * International Maize and Wheat Improvement Center (CIMMYT), Apdo. Postal 6-641, 06600, Mexico DF, Mexico; † Colegio de Postgraduados, Montecillos, Edo. de Mexico, 56230, Mexico; ‡ The Roslin Institute, University of Edinburgh, Easter Bush, Midlothian, EH25 9RG, United Kingdom; § Department of Biostatistics, School of Public Health, University of Alabama at Birmingham, Alabama 35294; ** Saaten Union Recherche, 163 Avenue de Flandre, 60190 Estrées Saint Denis, France; †† USDA—ARS, Department of Plant Breeding and Genetics, Cornell University, Ithaca, New York, New York 14850

**Keywords:** GenPred, shared data resources, genotyping by sequencing, genomic selection, imputation, GBLUP, RKHS

## Abstract

Genotyping-by-sequencing (GBS) technologies have proven capacity for delivering large numbers of marker genotypes with potentially less ascertainment bias than standard single nucleotide polymorphism (SNP) arrays. Therefore, GBS has become an attractive alternative technology for genomic selection. However, the use of GBS data poses important challenges, and the accuracy of genomic prediction using GBS is currently undergoing investigation in several crops, including maize, wheat, and cassava. The main objective of this study was to evaluate various methods for incorporating GBS information and compare them with pedigree models for predicting genetic values of lines from two maize populations evaluated for different traits measured in different environments (experiments 1 and 2). Given that GBS data come with a large percentage of uncalled genotypes, we evaluated methods using nonimputed, imputed, and GBS-inferred haplotypes of different lengths (short or long). GBS and pedigree data were incorporated into statistical models using either the genomic best linear unbiased predictors (GBLUP) or the reproducing kernel Hilbert spaces (RKHS) regressions, and prediction accuracy was quantified using cross-validation methods. The following results were found: relative to pedigree or marker-only models, there were consistent gains in prediction accuracy by combining pedigree and GBS data; there was increased predictive ability when using imputed or nonimputed GBS data over inferred haplotype in experiment 1, or nonimputed GBS and information-based imputed short and long haplotypes, as compared to the other methods in experiment 2; the level of prediction accuracy achieved using GBS data in experiment 2 is comparable to those reported by previous authors who analyzed this data set using SNP arrays; and GBLUP and RKHS models with pedigree with nonimputed and imputed GBS data provided the best prediction correlations for the three traits in experiment 1, whereas for experiment 2 RKHS provided slightly better prediction than GBLUP for drought-stressed environments, and both models provided similar predictions in well-watered environments.

Implementation of genomic selection (GS) in plant and animal breeding programs is usually based on genotypes derived from marker panels discovered using reference samples. The rapid progress of next-generation DNA sequencing (NGS) technologies, especially those based on short read sequencing of a fractional genome representation, have allowed direct and inexpensive single nucleotide polymorphism (SNP) detection from large and diverse germplasm collections. Genotyping-by-sequencing (GBS) is an NGS approach that reduces genome complexity via restriction enzymes (Elshire *et al.* 2011). GBS has been applied to study trait association in a diverse seed bank collection of maize germplasm (Romay *et al.* 2013) to identify agriculturally important but uncommon alleles (Yan *et al.* 2010). GBS has also been used to construct GS models for large and complex polyploidy wheat breeding materials (Poland *et al.* 2012).

One biological complication of applying GBS in a maize breeding program is that maize possesses a dynamic genome with extensive presence–absence variation (Fu and Dooner 2002), which results in many regions of the genome not being imputable for a given SNP. Recent studies suggest 80–90% of the genome shows some presence–absence variation (Chia *et al.* 2012). High-depth coverage studies using GBS suggest that only 75–82% of the sites are present in these samples, except for the reference genome B73, which exhibits near-complete coverage.

GBS data are highly dimensional and usually come with a large percentage of uncalled genotypes; therefore, incorporating this information into models poses important statistical and computational challenges that need to be addressed but have not yet been studied in detail. First, with low sequencing coverage, the proportion of “missing” marker genotypes can be high (∼70–80% or more for 1× sequencing coverage; Poland *et al.* 2012), and it is not clear how massive amounts of GBS information can be incorporated into statistical models for prediction of performance of unobserved genotypes (as required in GS). Second, it is unknown how GBS data information can be combined with pedigree information in model prediction. Third, until now, it is not known how imputation of missing genotype data or haplotype inferences may affect prediction accuracies when GBS data are used.

Haplotypes can be used to impute missing data; to perform this imputation, only a few individuals need to carry a given haplotype in the data set. However, many more copies of a given haplotype are required for an accurate statistical estimation of its effect. Longer haplotypes may be more accurately determined, but by nature they are carried by fewer individuals, especially when the sample size is small. Longer haplotypes can directly parameterize presence–absence variants or other types of QTL alleles nested within haplotype alleles, but SNP markers need to parameterize them indirectly through linkage disequilibrium (LD). It is not known how the imputation and prediction with different haplotypes lengths affect genomic prediction.

Most GS research so far has focused on evaluation based on low-to-intermediate marker panels with a low-to-intermediate proportion of missing markers (de los Campos *et al.* 2009, 2010; Crossa *et al.* 2010, 2011; Pérez *et al.* 2010, 2012; González-Camacho *et al.* 2012; Heslot *et al.* 2012; Hickey *et al.* 2012). GBS has attracted great interest for the application of GS in plant breeding. Poland *et al.* (2012) recently reported that they successfully applied GBS to a large set of advanced wheat lines from the semi-arid wheat breeding program of CIMMYT and predicted breeding values with 0.28 to 0.45 prediction accuracy for grain yield (GY), an improvement of 0.1 to 0.2 over another marker platform. The authors concluded that its low cost makes GBS a good method for GS. The extensive LD of most wheat populations probably aids the accuracy of imputation of missing genotype data. However, the accuracy of genomic predictions based on imputed data may be quite different in other species, such as maize, with much less extensive LD. Although GBS appears promising as a genotyping method to use in GS, currently, no studies have been performed that evaluate the use of GBS data for prediction accuracy in diverse maize breeding populations.

Therefore, the main objective of this research was to quantify the accuracy of genomic prediction using GBS data in two maize data sets with different complex (or quantitative) traits evaluated under differing environmental conditions. Experiment 1 included three traits evaluated in four environments, as well as pedigree information regarding the maize lines. Experiment 2 included one trait and two environments, but no information regarding the pedigree of the maize lines. The accuracy of genomic predictions using GBS was assessed by examining the prediction accuracy of three different sources of GBS information: nonimputed GBS; imputed GBS; and GBS-inferred haplotypes. In experiment 1, pedigree data were available; therefore, in this data set, we were able to assess how much prediction accuracy can be gained by combining pedigree and GBS data, relative to family-based predictions. Experiment 2 has been analyzed by other authors using SNP arrays; therefore, in this experiment, we were able to compare the prediction accuracy that can be attained with GBS data relative to previous reports using SNP arrays.

In one dimension of this study, the predictions of the different sources of GBS information are compared. In another dimension, we examine the prediction accuracy of two statistical models: genomic best linear unbiased predictor (GBLUP) and reproducing kernel Hilbert spaces (RKHS) for all three types of GBS information. The last dimension evaluated in this study, which applies only to experiment 1 (with a family structure), is concerned with the prediction accuracy of GBLUP and RKHS including or ignoring pedigree information in combination with the three types of GBS information. A default GBS-inferred haplotype length consisting of 1000 flanking markers on each side of the marker being imputed was used for both experiments. For experiment 2 (with a smaller sample size than experiment 1), we used two other haplotype lengths, short and long, with 50 and 100 flanking markers on each side of the marker being imputed, respectively.

## Materials and Methods

Two compressed files containing phenotypic and genotypic information regarding the two data sets used in this study [experiment 1 with 504 maize doubled haploid (DH) lines and experiment 2 with 296 maize lines] can be downloaded from http://repository.cimmyt.org/xmlui/handle/10883/1380.

### Phenotypic data

The phenotypic data set consisted of two different maize data sets, experiment 1 and experiment 2. The data set from experiment 1 was a collection of 504 DH lines derived from crossing and backcrossing eight inbred parents to form 10 full-sib families. The 504 DH were crossed to a single-cross tester. Basic information regarding the parents in experiment 1 is provided in Table A1 (see Appendix A). Data from experiment 2 consisted of a diverse panel of 296 CIMMYT maize inbred lines, including several breeding populations.

**Table A1:** Number of doubled-haploid lines in each cross (P1×P2) and backcross, and pedigree of the eight parents (A, B, C, D, E, F, G, and H) included in experiment 1

Size	P1	P2	BC	Parent	Pedigree
64	A	B	B	A	La Posta Seq C7-F96-1-2-1-1-B-B-B-B
78	A	C	C	B	CML395 (IITA)
29	A	D	D	C	CML444 (population 43 cycle 9)
37	E	F	F	D	CML488 (CIMMYT-ZIMBABWE line)
108	E	B	B	E	La Posta Seq C7-F71-1-2-1-2-B-B-B-B
83	E	C	C	F	MAS[MSR/312]-117-2-2-1-B*4-B-B-B
29	E	D	D	H	La Posta Seq C7-F102-1-3-1-2-B-B-B-B
49	G	B	B	G	[M37W/ZM607#bF37sr-2-3sr-6-2-X]-8-2-X-1-BB-B-xP84c1 F27-4-3-3-B-1-B] F29-1-2-2 × [KILIMA ST94A]-30/MSV-03-101-08-B-B-1xP84c1 F27-4-1-4-B-3-B] F2-1-2-1-1-1-B × CML486]-1-1-B
9	H	B	B		
18	H	D	D		

#### Phenotypic data for 504 DH maize lines (experiment 1):

A total of 504 DH maize lines were obtained by crossing and backcrossing eight parents to form 10 full-sib families of different sizes. Each DH line was crossed to an elite single-cross hybrid from the opposite heterotic group to produce 504 test-crosses that were genotyped with GBS. Traits available in this data set included GY (kg/ha), anthesis date (AD; days after sowing), and anthesis-silking interval (ASI; days); each of these traits were evaluated in four environments, three optimum rain-fed trials and one managed drought trial. The experimental design in each of the four environments was an alpha-lattice incomplete block design with two replicates.

Although individual trial prediction analyses were performed for the three optimum and one drought environment, here we present results for the four trials combined. Data were balanced across the four trials and pre-adjusted using estimates of block and environment effects derived from a linear model that accounted for the incomplete block design within environment and for environment effects. Given that these data had a family structure, pedigree information was incorporated into the prediction in two forms, as a pedigree model *per se* and by including it in the GBLUP and RKHS models.

Broad-sense heritability of the combined analyses across environments was calculated as$h^{2}=\sigma_{g}^{2}/(\sigma_{g}^{2}+\frac{\sigma_{ge}^{2}}{e}+\frac{\sigma_{e}^{2}}{e\times r})$, where $\sigma_{g}^{2}$, $\sigma_{ge}^{2}$, and $\sigma_{e}^{2}$ are the genotype, genotype × environment interaction, and error variance components, respectively, and *e* and *r* are the number of environments and replicates within each environment included in the corresponding analyses, respectively.

#### Phenotypic data for the 296 inbred lines (experiment 2):

These lines represent a wide range of germplasm from the global maize breeding program of CIMMYT and were derived from different breeding subpopulations. The two trait combinations considered in this study were GY (kg/ha) evaluated in four severe drought stress (SS) trials and five well-watered (WW; ample rainfall) trials. All 296 lines were included in all the trials and pre-adjusted based on estimates of block and plot effects derived from the linear model, as described for data in experiment 1; broad-sense heritability of the combined analyses across environments was calculated as explained in experiment 1. This data set was also used by Crossa *et al.* (2010), González-Camacho *et al.* (2012), and Windhausen *et al.* (2012).

### Genotypic data

#### Genotyping-by-sequencing:

GBS is a reduced representation approach that uses restriction enzymes to sample the genome. In this study, we adopted a GBS protocol commonly used by the maize research community (Elshire *et al.* 2011; Romay *et al.* 2013). DNA was digested with *Ape*KI restriction enzyme and 96 samples were multiplexed per Illumina flow cell for sequencing.

To increase the genome coverage and read depth for SNP discovery, raw read data from sequencing the samples were analyzed together with 19,000 additional maize samples from the CIMMYT global maize breeding program, USA and Chinese NAM populations, and the USDA Ames maize collection and teosinte mapping populations. SNP identification was performed using TASSEL 3.0 GBS Discovery Pipeline with B73 as the reference genome (http://www.maizegenetics.net/tassel/docs/TasselPipelineGBS.pdf). Briefly, raw Illumina DNA sequence data (∼100 base pair qseq file) were first trimmed to remove bar codes, and further trimmed or padded with “A's on the 3′ end to 64 base pair lengths. First, the SNP calling process was initiated by identifying all distinct 64-base pair tags occurring in more than 0.1% of the overall maize collection (∼20,000 lines). Second, the distribution of these tags across all *Zea* was then generated, and tags were aligned against the reference genome sequence of B73 using the Burrows-Wheeler aligner (Li and Durbin 2009). Third, all tags aligning in the same region of the genome are aligned with the full multiple sequence alignment of Biojava3 (http://biojava.org/) (Holland *et al.* 2008). Finally, a SNP was defined for every maize sample based on a binomial distribution from the aligned unique tags.

Because of extensive paralogy in maize, SNPs were then further filtered based on homozygosity in inbred lines, the biparental error correction plug-in in Tassel GBS Discovery Pipeline, which was applied to eliminate SNP with high errors rates (−m × E = 0.01), and a minimum median *r^2^* (parameter: −mnPLD = 0.5) for LD with markers in the local genome region across biparental families. And, finally, only those that segregate in the mapping populations were used in this study. Source code and the Tassel GBS discovery pipeline are available at www.maizegenetics.net and SourceForge Tassel project (http://sourceforge.net/projects/tassel/). Tassel was also used for computing pairwise measures of LD (*r*^2^) between loci for each data set (experiments 1 and 2) and for each chromosome within the data sets (Bradbury *et al.* 2007).

### Imputation and haplotype methods

A simple imputation algorithm was developed for the purpose of imputing the markers that were missing because of technical limitations with GBS data (not biologically missing). The imputation algorithm borrowed ideas related to haplotype library imputation (Hickey *et al.* 2012). In the variant of the algorithm used in this study, imputation works by first identifying individuals who are likely to share a common haplotype. Then, the genotype information from these individuals is combined to form a consensus haplotype. Finally, any individuals who share this haplotype have their missing markers imputed on the basis of this consensus haplotype.

The individuals used in this study were inbred lines and therefore were homozygous for the majority of markers. The imputation procedure began by first setting to missing any residual heterozygote markers. Next, diploid genotype data were formally phased based on the fact that phase could be directly observed, because markers not originally missing or set to missing were phased *de facto* by virtue of the genotypes being homozygous.

Imputation was performed at each marker using information from flanking markers, both called and missing, to define the haplotype allele that each individual carried. At each marker, the genotyped individuals in the data set were partitioned into clusters, and individuals in the same cluster were assumed to carry the same haplotype because they had no opposing homozygote loci. This was performed by searching a certain number of flanking markers on either side of the marker of interest for called alleles that disagreed between pairs of individuals. Pairs of individuals with more than a threshold with four disagreeing alleles were assumed not to belong to the same cluster. Individuals belonged to only one cluster, and the minimum number of individuals in a cluster was one. Individuals in the same cluster were assumed to carry the same haplotype and, therefore, the same allele at the marker of interest. If some individuals in the cluster were missing this allele, but others were not, then it was assumed that the allele was technically missing (rather than biologically missing) in individuals in this cluster who were missing the allele. The missing allele was therefore imputed in those individuals in whom it was missing. If the allele was missing for all individuals in a cluster, then it was not imputed because it was assumed to be biologically missing for these individuals. The algorithm moved along each chromosome, moving the locus to be imputed and its flanking markers, one marker at a time.

#### Haplotype length:

The imputation algorithm that was performed at each marker uses information from flanking markers (that are called or missing) and defines the haplotype allele that each individual carries. Therefore, the algorithm imputes not only technically missing markers but also inferred haplotypes that can be used for genomic prediction. Explicitly, the inferred haplotypes are, in fact, the clusters to which each individual belongs.

In the analysis of both data sets, the default imputation method used 1000 flanking markers on each side of the marker being imputed to infer the haplotype allele carried. However, because of the small size of the data set in experiment 2, the long default haplotypes would not be informative because too few individuals were present for enough individuals to share long haplotypes; therefore, two other parameter values for the imputation method were used to examine the sensitivity of haplotypes length, one using a short haplotype inference from 50 flanking markers on each side of the marker being imputed and another using a long haplotype inference from 100 flanking markers. The haplotype lengths were chosen as a compromise between computation time and the overall yield of imputed alleles in some cases and imputation accuracy in other cases. Shorter haplotypes take more time and generate a greater amount of imputed alleles. Longer haplotypes have a greater accuracy of imputation, but if they are too long they are shared by very few individuals and consequently contribute fewer imputed alleles.

Clusters that were created and used to perform the imputation also contained information that could be used for genomic prediction directly. Individuals belonging to the same cluster at each marker position were assumed to carry the same haplotype at that marker position. Because there were several clusters at each marker position, there were also several haplotypes at each marker position. These clusters/haplotypes may be able to parameterize biologically missing data more optimally than partially imputed SNP. For example, an allele that is missing for all individuals in a cluster is not directly parameterized by SNP because all individuals in this cluster will be missing for this SNP. However, the cluster/haplotype alleles may be able to more directly parameterize this because all individuals carry one of the haplotype alleles, and the biologically missing variant may be nested within only one or a few haplotype alleles. Longer haplotypes may be more precise in their definition of the haplotype allele carried by an individual; however, by virtue of their length, long haplotypes are not shared by as many individuals as short haplotypes. Therefore, because more copies of each haplotype allele are present in the data for short haplotypes, short haplotypes may be more powerful for genomic prediction.

In essence, there are multiple tradeoffs involved. Longer haplotypes are more precisely defined, but alleles may not be shared by many individuals and therefore have reduced power for estimating their effects. Furthermore, long haplotypes may have greater ability to capture nonadditive genetic effects or the effects of recent mutations that are more likely to be shared by very close relatives. Short haplotypes and SNPs may have greater ability to parameterize additive effects caused by older mutations.

### Statistical models

In an applied breeding program, GS could be applied for two purposes: predicting breeding values of individuals for rapid selection cycling or predicting genotypic values of advanced lines that are in the last stages of testing. Although predicting breeding values necessitates models for estimating additive effects, predicting the genetic values of advanced lines requires models that account for additive as well as nonadditive (*i.e.*, epistatic) genetic effects (Crossa *et al.* 2013). In general, linear models such as GBLUP capture additive relationships, whereas nonlinear models such as RKHS can account for nonadditive genetic components (*e.g.*, gene × gene effects) (Gianola *et al.* 2006; de los Campos *et al.* 2010; González-Camacho *et al.* 2012).

Genomic prediction (Meuwissen *et al.* 2001) can be implemented using parametric or semi-parametric procedures. Among the parametric methods, GBLUP (a ridge-regression–type estimator) is the most commonly used; among the semi-parametric procedures, RKHS is the one used most frequently (*e.g.*, de los Campos *et al.* 2012) and has consistently shown high prediction accuracy (*e.g.*, de los Campos *et al.* 2010; Crossa *et al.* 2010, 2011; Heslot *et al.* 2012; González-Camacho *et al.* 2012; Pérez *et al.* 2012). In this study, we considered using either GBLUP or RKHS regressions.

The GBLUP method uses a genomic relationship matrix computed using imputed or nonimputed marker information. Furthermore, the genomic relationship matrix can also be computed on a SNP-by-SNP basis or on the basis of haplotypes spanning multiple adjacent SNP (see Appendix A for further details). Haplotypes can be used instead of imputed GBS markers when constructing genomic relationship matrices for GBLUP or the reproducing kernel for RKHS regressions (further details are provided in Appendix A).

The RKHS implementation applied here uses a Gaussian kernel evaluated on Euclidean distances computed from either imputed or nonimputed GBS data or from SNP *vs.* haplotype information. We implemented the RKHS regression using a multi-kernel approach, termed kernel averaging (KA), described by de los Campos *et al.* (2010). Further details regarding our implementation are given in Appendix A. For the data set with available pedigree information (504 DH lines), we evaluated the GBLUP and RKHS models using genomic information and a combination of genomic and pedigree information.

We implemented GBLUP and RKHS using the Bayesian framework by assigning appropriate prior probability distributions to marker effects (see Appendix A). The posterior distributions of all unknowns in both models were obtained using MCMC techniques. We used the Bayesian implementation of RKHS described by de los Campos *et al.* (2010) with the kernels and prior probability distributions described in González-Camacho *et al.* (2012).

#### Notation of the different models:

Concerning the notation of the models, we appended to either GBLUP or RKHS subscripts that denote the imputation and haplotype methods used. No subscripts were used for nonimputed GBS data. For experiments 1 and 2 data sets, the default marker imputation method (1000 flanking markers) is denoted by the subscript IM (GBLUP_IM_, RKHS_IM_); for the haplotype-based method, we used H as the subscript (GBLUP_H_, RKHS_H_). For experiment 1, the pedigree model denoted as P is fitted as described in Appendix A and when combined pedigree and marker information is used with nonimputed GBS, the notation is PGBLUP and PRKHS. When imputed (IM) GBS and haplotype-based (H) GBS methods are used, the notations are PGBLUP_IM_, PRKHS_IM_, PRKHS_H_, and PRKHS_H_, respectively.

For experiment 2, when imputed marker data are from short haplotypes (50 flanking markers; IMS) and long haplotypes (100 flanking markers; IML), the models are denoted as GBLUP_IMS_, GBLUP_IML_, RKHS_IMS_, and RKHS_IML_, respectively, and the models that use the inferred short haplotypes (HS) and long haplotypes (HL) are named GBLUP_HS_, GBLUP_HL_, RKHS_HS_, and RKHS_HL_, respectively. Further details regarding the computation of GBLUP and RKHS are provided in Appendix A.

#### Cross validation scheme:

We quantified prediction accuracy using a replicated training–testing evaluation. In each replicate, data were randomly partitioned into training (TRN; with 70% of the observations) and testing (TST; with 30% of data points) data sets. Models were fitted to the TRN data set and prediction accuracy was evaluated in the TST data set. This was replicated 50 times with independent random assignments into TRN and TST; in this manner, we obtained 50 estimates of the prediction accuracy (one per replicate) for each of the methods evaluated. Prediction ability was quantified using the correlation between predicted and observed values in each of the TST data sets. A scheme similar to this was used by Gianola *et al.* (2011) and González-Camacho *et al.* (2012); one advantage of this approach is that in addition to yielding a point estimate of prediction accuracy, it provides measures of uncertainty of such estimates (*e.g.*, variance of predictive correlation across partitions of the data into TRN and TST data sets).

## Results

For each data set, results are presented in relation to general information on the GBS data as well as information on GBS data in each experiment data set (number of missing SNPs by chromosome and distribution of minor allele frequency), and in relation to the prediction accuracy of the two statistical models (GBLUP and RKHS) on the three different types of GBS information (imputed, nonimputed, and haplotype method). For experiment 1, results of the prediction accuracy of GBS for models with and without pedigree are examined.

### GBS data

Obtained from a large collection of maize samples (∼20,000 lines), approximately 66% of the unique reads mapped to unique positions in the reference genome; if mapped uniquely, then 80% of the 64 base pair reads carried at least one polymorphic site among all varieties. This is the result of sampling a large collection of diverse material, in which 45% of the SNP sites are polymorphic, plus more from small indels; the rest of the SNPs (∼55%) are classified as rare alleles (with very low allele frequency). On average, 2.09 billion single base pair reads can be collected in a single flow cell. At the plex level of 96, the coverage of GBS SNPs in this study is approximately at 1×, given the averaged raw read counts for our maize samples being 2.3 million reads. However, sequence read depth varies greatly across the genome, especially when samples are run from 1× to 2× coverage (Beissinger *et al.* 2013); genome coverage also varies by taxa and SNP sites, and thus it has been complicated to systematically describe the read depth variation along the genome and across taxa. At 1×, the Poisson distribution expects 36.8% of missing information. Our data in Table 1 and Table 2 suggest GBS has a higher than expected missing data ratio.

**Table 1:** Experiment 1 (504 doubled-haploid maize lines), initial number of markers per chromosome, number of filtered markers per chromosome, and percentage of missing cells per chromosome after imputation

Chromosome	Initial Number of Markers (% of Missing Cells Before Filter)	Number of Markers After Filter (% of Missing Cells After Filter)	% Missing Cells After Imputation
1	108,292 (51.3)	23,878 (43.37)	19.67
2	82,006 (52.5)	20,191 (44.63)	20.95
3	77,207 (51.9)	18,360 (44.02)	20.52
4	66,512 (52.8)	13,814 (44.58)	20.48
5	77,067 (51.8)	17,784 (43.78)	19.78
6	55,489 (52.4)	13,893 (44.23)	20.84
7	57,504 (52.6)	13,354 (44.52)	20.82
8	58,568 (52.0)	14,903 (43.99)	20.67
9	50,473 (52.2)	10,823 (43.30)	19.53
10	48,139 (52.7)	11,281 (44.72)	20.76
Total	681,257	158,281	

Filter of markers consisted of deleting markers that had >80% of lines with missing values and deleting markers whose minor allele frequency was ≤0.05.

**Table 2:** Experiment 2 (296 maize lines), initial number of markers, number of filtered markers per chromosome, percentage of missing cells in the default imputation method, imputation method for short haplotypes, and imputation method for long haplotypes

Chromosome	Initial Number of Markers (% of Missing Cells Before Edit)	Number of Edited Markers (% of Missing Cells After Filter)	% Missing Cells After the Default Imputation	% Missing Cells After Imputation Method for Short Haplotypes	% Missing Cells After Imputation Method for Long Haplotypes
1	108,292 (57.44)	37,376 (51.66)	48.71	24.58	33.65
2	82,006 (58.27)	29,283 (52.23)	49.38	25.87	33.95
3	77,207 (58.06)	27,287 (51.85)	48.75	24.93	33.95
4	66,512 (58.80)	20,672 (51.87)	49.27	25.32	33.96
5	77,067 (57.72)	27,453 (51.80)	48.70	25.27	33.14
6	55,489 (58.27)	19,650 (51.92)	48.72	25.03	33.71
7	57,504 (58.40)	19,896 (52.33)	49.55	25.66	32.99
8	58,568 (58.18)	20,248 (51.80)	48.03	24.87	32.67
9	50,473 (58.27)	17,027 (51.95)	49.24	25.14	33.40
10	48,139 (58.84)	16,373 (52.65)	48.95	24.47	33.46
Total	681,257	235,265			

Filter of markers consisted of deleting markers that had >80% of lines with missing values and deleting markers whose minor allele frequency was ≤0.05.

Based on the repeated samples in this large maize collection (Romay *et al.* 2013), the error rate per base was estimated at 0.0018 based on repeated samples, and the average SNP call rate (per sample) was 42%, with values ranging from 2% to 75%. However, when analyzing taxa that have deeper coverage, an average of 16–23% of the sites are missing from any given sample, suggesting a substantial amount of missing data are attributable to the biology of presence/absence variation in the maize genome (Springer *et al.* 2009).

Information on genotypic data for each maize chromosome, including the number of original markers, the percentage of missing cells (where cell refers to the value of a locus in an individual line) in the original marker data, the number of markers after initial filtering, and the number of markers after imputation, is summarized in Table 1 and Table 2 for experiments 1 and 2, respectively. Filtering was performed by first removing markers that had more than 80% of lines with missing values and then deleting markers with MAF ≤0.05. Overall, before filtering, we obtained a total of 681,257 SNP directly from the GBS calling pipeline; after filtering for missing values and minor allele frequency, 158,281 SNPs were used in experiment 1 (Table 1) and 235,265 SNPs were used in experiment 2 (Table 2).

**Figure 1:**
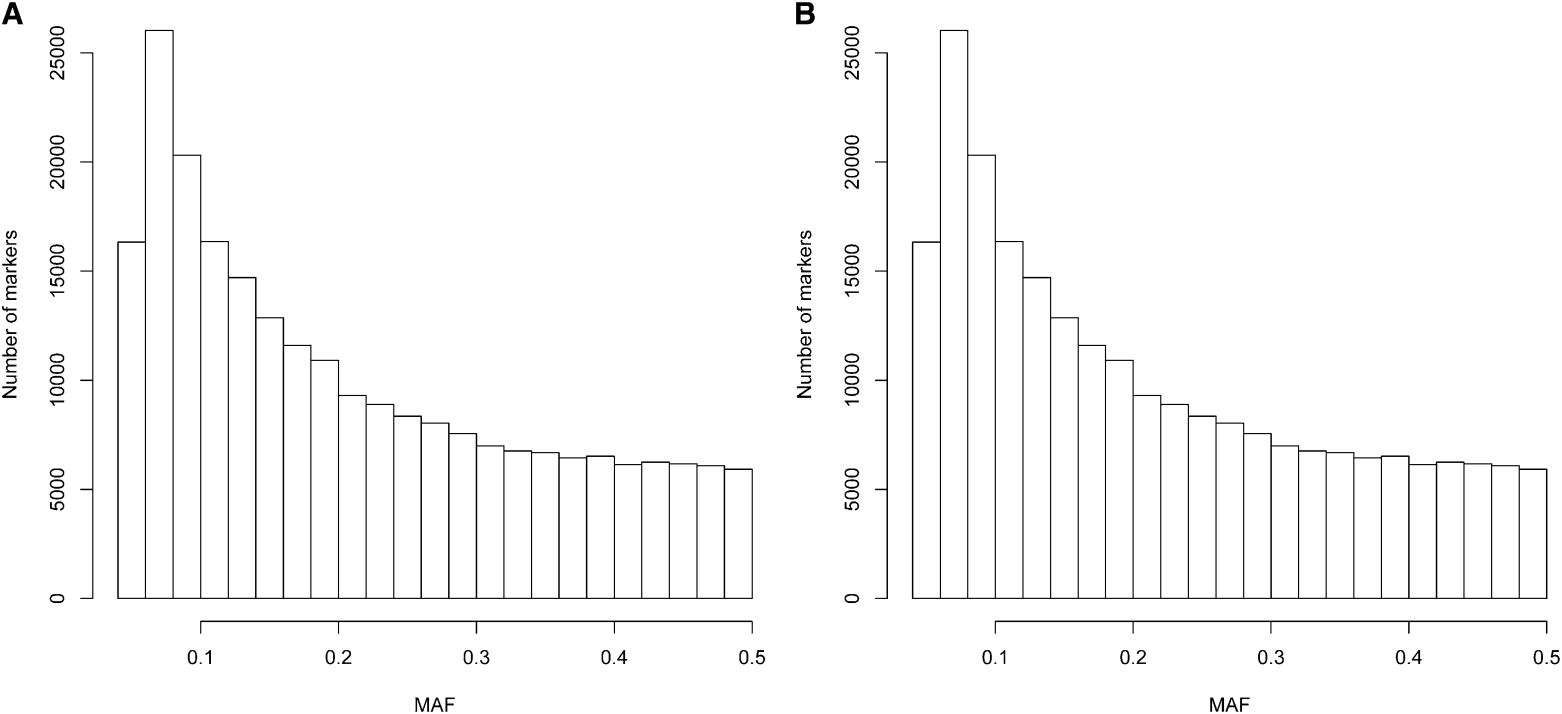
(A) Distribution of minor allele frequency (MAF) for experiment 1. (B) Distribution of minor allele frequency for experiment 2. Data used is non-imputed genotyping-by-sequencing (GBS).

For experiment 1 (Table 1), the initial GBS data before filtering had a percentage of missing cells per chromosome ranging from 51.3 to 52.8%; after filtering, this percentage decreased to approximately 43–44% of the total number of cells. Approximately 20% of cells were missing in the edited GBS information used for prediction after imputation (last column in Table 1). These 20% missing genotypes were replaced by their expected values before performing the prediction. The distribution of minor allele frequency is depicted in Figure 1A. Regarding GBS experiment 2 (Table 2), the initial GBS markers for this data set had approximately 57–58% of missing cells. The percentage of missing cells in the edited GBS data was 51–52% (Table 2), and minor allele frequency distribution is shown in Figure 1B.

**Figure B1:**
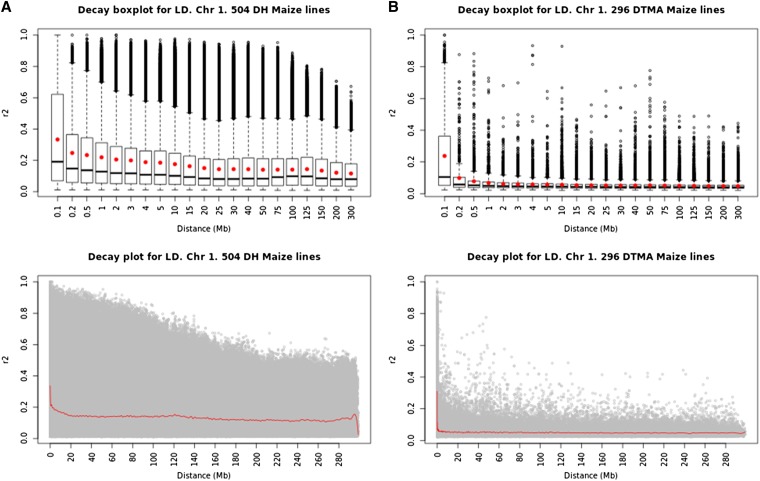
(A and B) Graphs of the box plot of *r^2^* and linkage disequilibrium decay at different marker distances in the (A) 504 DH maize lines (experiment 1) and (B) 296 maize lines (experiment 2). The *r^2^* and linkage disequilibrium decay are shown at different marker distances for chromosome 1.

**Figure B2:**
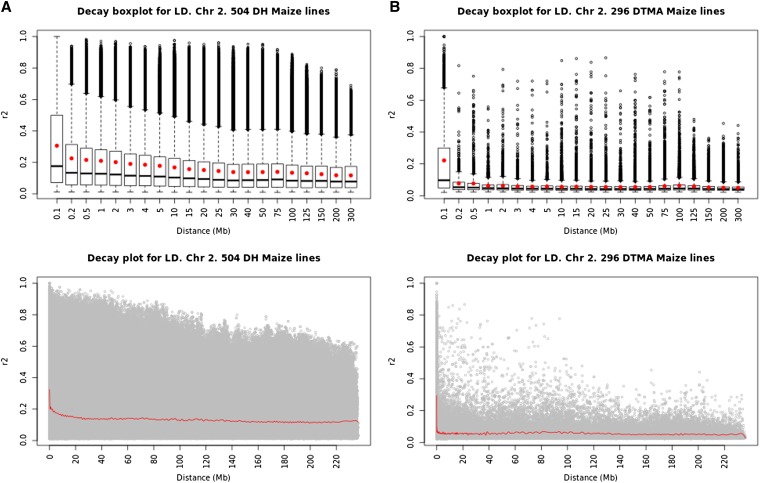
(A and B) Graphs of the box plot of *r^2^* and linkage disequilibrium decay at different marker distances in the (A) 504 DH maize lines (experiment 1) and (B) 296 maize lines (experiment 2). The *r^2^* and linkage disequilibrium decay are shown at different marker distances for chromosome 2.

**Figure B3:**
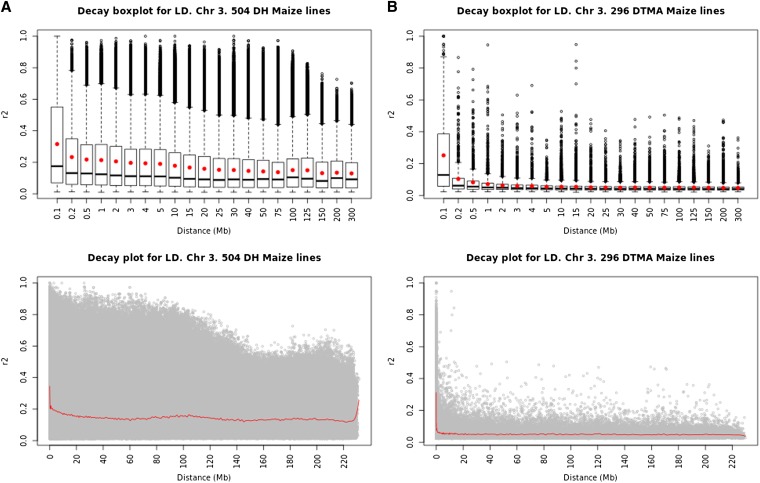
(A and B) Graphs of the box plot of *r^2^* and linkage disequilibrium decay at different marker distances in the (A) 504 DH maize lines (experiment 1) and (B) 296 maize lines (experiment 2). The *r^2^* and linkage disequilibrium decay are shown at different marker distances for chromosome 3.

**Figure B4:**
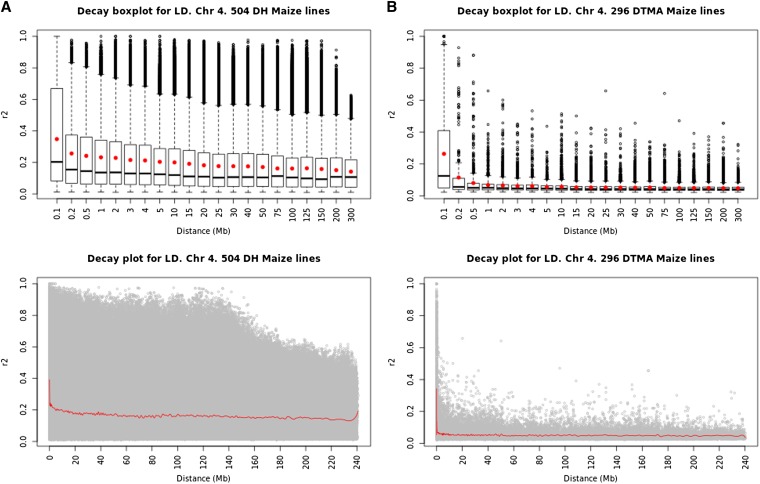
(A and B) Graphs of the box plot of *r^2^* and linkage disequilibrium decay at different marker distances in the (A) 504 DH maize lines (experiment 1) and (B) 296 maize lines (experiment 2). The *r^2^* and linkage disequilibrium decay are shown at different marker distances for chromosome 4.

**Figure B5:**
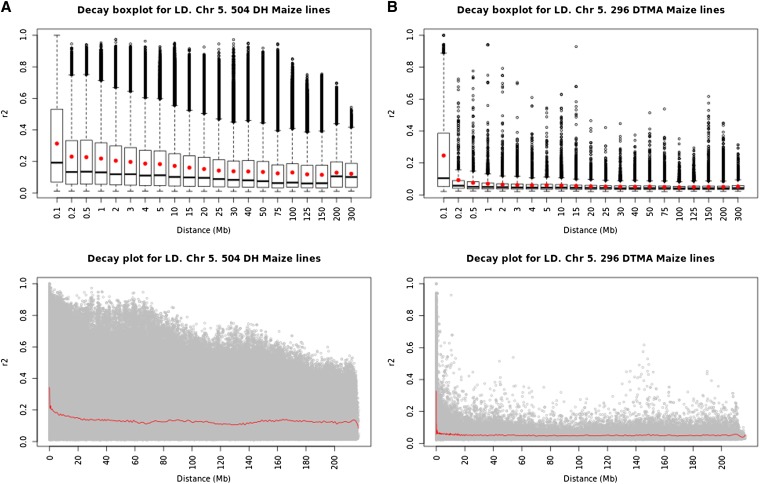
(A and B) Graphs of the box plot of *r^2^* and linkage disequilibrium decay at different marker distances in the (A) 504 DH maize lines (experiment 1) and (B) 296 maize lines (experiment 2). The *r^2^* and linkage disequilibrium decay are shown at different marker distances for chromosome 5.

**Figure B6:**
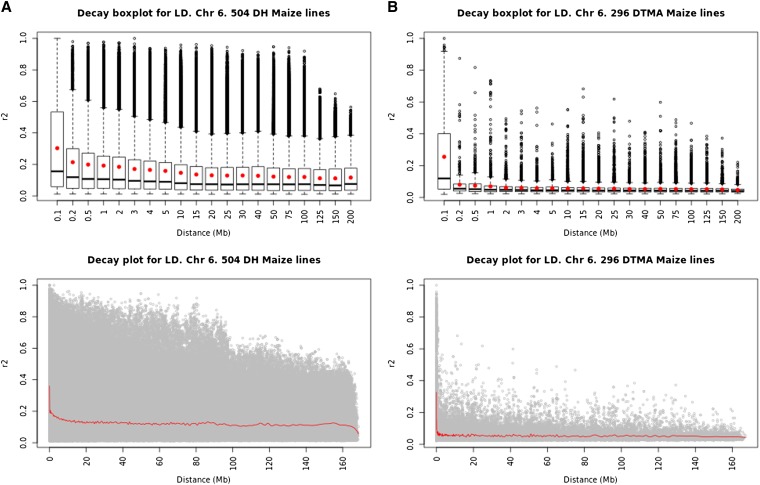
(A and B) Graphs of the box plot of *r^2^* and linkage disequilibrium decay at different marker distances in the (A) 504 DH maize lines (experiment 1) and (B) 296 maize lines (experiment 2). The *r^2^* and linkage disequilibrium decay are shown at different marker distances for chromosome 6.

**Figure B7:**
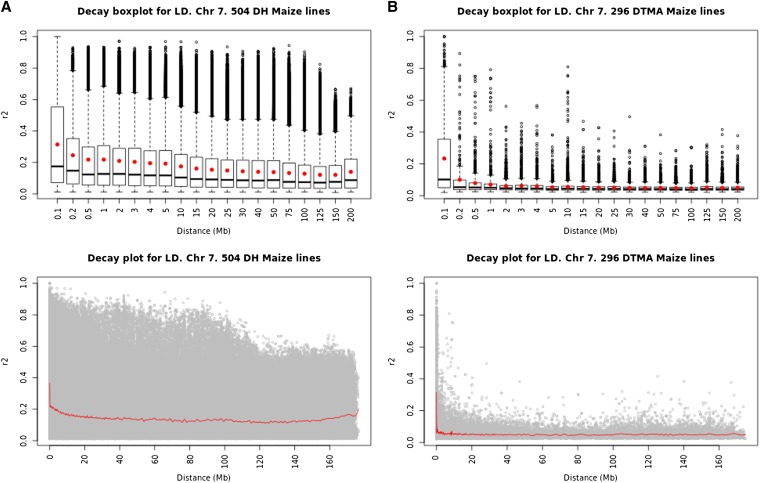
(A and B) Graphs of the box plot of *r^2^* and linkage disequilibrium decay at different marker distances in the (A) 504 DH maize lines (experiment 1) and (B) 296 maize lines (experiment 2). The *r^2^* and linkage disequilibrium decay are shown at different marker distances for chromosome 7.

**Figure B8:**
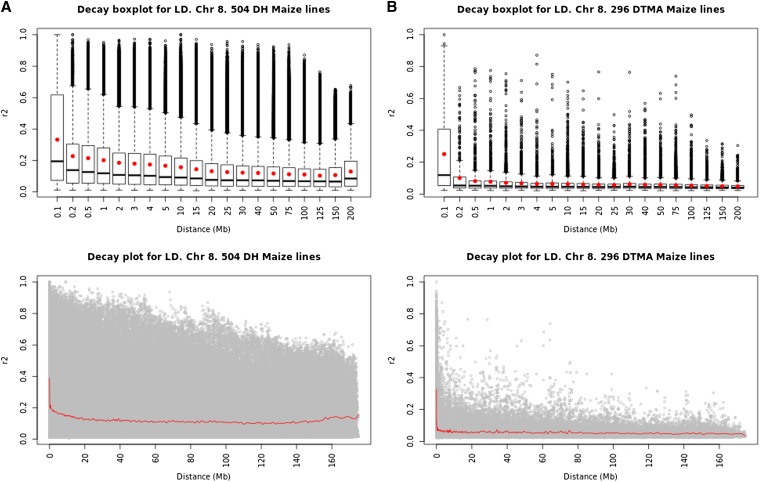
(A and B) Graphs of the box plot of *r^2^* and linkage disequilibrium decay at different marker distances in the (A) 504 DH maize lines (experiment 1) and (B) 296 maize lines (experiment 2). The *r^2^* and linkage disequilibrium decay are shown at different marker distances for chromosome 8.

**Figure B9:**
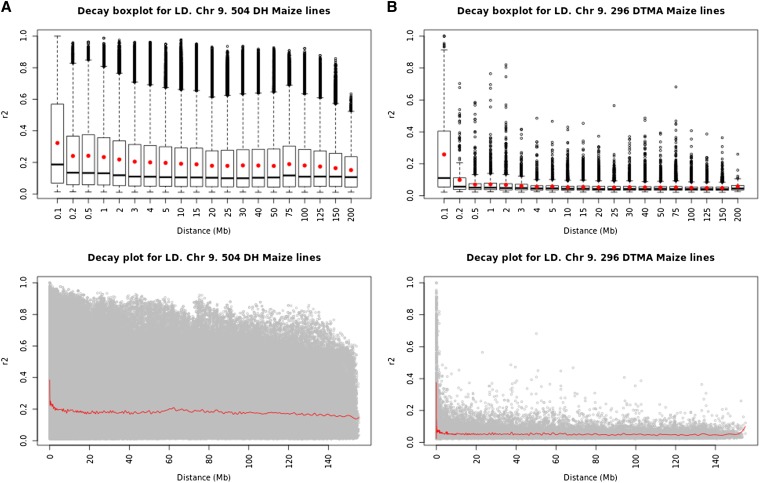
(A and B) Graphs of the box plot of *r^2^* and linkage disequilibrium decay at different marker distances in the (A) 504 DH maize lines (experiment 1) and (B) 296 maize lines (experiment 2). The *r^2^* and linkage disequilibrium decay are shown at different marker distances for chromosome 9.

**Figure B10:**
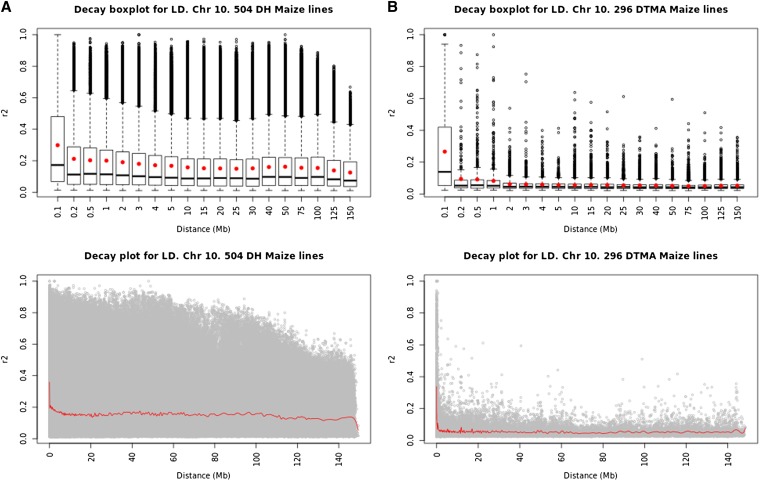
(A and B) Graphs of the box plot of *r^2^* and linkage disequilibrium decay at different marker distances in the (A) 504 DH maize lines (experiment 1) and (B) 296 maize lines (experiment 2). The *r^2^* and linkage disequilibrium decay are shown at different marker distances for chromosome 10.

Measures of LD at various genetic distances by data set and chromosome were computed. Plots of *r*^2^ *vs.* distance by chromosome and data set are depicted in Appendix B (Figure B1, a and b; Figure B2, a and b; Figure B3, a and b; Figure B4, a and b; Figure B5, a and b; Figure B6, a and b; Figure B7, a and b; Figure B8, a and b; Figure B9, a and b; and Figure B10, a and b). The *r*^2^ between adjacent markers decreased very quickly in experiment 2, as expected for maize, and the median *r*^2^ achieved very low values at distances of 0.5 Mb or longer. The patterns of LD in experiment 1 were very different; here, the average *r*^2^ remained relatively high (values of ∼0.2) even at very long distances, and there was great deal of variability in *r*^2^ even at long distances. This occurs because the association of alleles in this data set is largely driven by family linkage, whereas in experiment 2 the association patterns of alleles are dominated by population LD.

### Imputation and haplotype

#### Imputation: GBS experiment 1:

For this data set, the default imputation method had 1000 flanking markers for deriving the haplotypes, creating several hundred thousand alleles per chromosome (*i.e.*, one SNP can be in different haplotypes and this is considered an allele). The information in Table 1 shows different information on markers and missing cells (where cell refers to the value of a locus in an individual line).

#### Imputation: GBS experiment 2:

As previously mentioned for experiment 2, in data sets with a smaller sample size than in experiment 1, the LD is likely to be lower than that in the experiment 1 data set, thus making the long default haplotypes (with 1000 flanking markers) much less informative. Therefore, for this data set, we tested the sensitivity of shortening the flanking markers by performing imputation with 100 flanking markers [long haplotype imputation method (IML) that produces long haplotypes (LH)] and with 50 flanking markers [short haplotype imputation method (IMS) that produces short haplotypes (SH)] and studied the prediction accuracy of these cases as well. The percentage of missing cells in the default imputation method was approximately 48–49%, whereas the imputation method using 50 flanking markers (SH) produced approximately 24–25% of missing cells, and the imputation method using 100 flanking markers (LH) had approximately 32–33% of the total number of missing cells replaced by their expected values before performing the prediction.

### Prediction accuracy

#### Prediction: experiment 1:

Estimates of broad-sense heritability and estimates of prediction accuracy for this experiment are provided in Table 3. Correlations for the pedigree (P) model were 0.518, 0.589, and 0.588 for GY, AD, and ASI, respectively. In general, GBLUP models showed lower predictive correlations than the RKHS regression models for the three traits. However, for all traits, PGBLUP and PRKHS had very similar prediction accuracies and showed consistently better predictive correlations than their corresponding counterparts without pedigree, GBLUP and RKHS. In several instances, GBLUP and RKHS showed lower prediction correlations than the pedigree model (*i.e.*, RKHS_H_ for GY and ASI, and all the GBLUPs for GY and ASI). For trait AD, the increase in prediction accuracy of all models with GBS information is remarkable as compared with the pedigree model.

**Table 3:** Experiment 1 (504 doubled-haploid maize lines), estimated broad-sense heritability and average predictive ability between observed and predicted values for grain yield, days to anthesis, and anthesis-silking interval for 13 models and their percent change relative to the pedigree model

Model	Grain Yield	Days to Anthesis	Anthesis-Silking Interval
Estimated broad-sense heritability	0.433	0.676	0.545
Average predictive correlation			
P	0.518 (0.045)	0.589 (0.033)	0.588 (0.046)
RKHS	0.553 (0.046)	0.711 (0.036)	0.591 (0.044)
RKHS_IM_	0.545 (0.045)	0.710 (0.035)	0.585 (0.044)
RKHS_H_	0.505 (0.050)	0.659 (0.040)	0.561 (0.046)
PRKHS	0.584 (0.044)	0.727 (0.032)	0.620 (0.043)
PRKHS_IM_	0.582 (0.045)	0.729 (0.031)	0.616 (0.043)
PRKHS_H_	0.548 (0.047)	0.681 (0.036)	0.598 (0.044)
GBLUP	0.469 (0.050)	0.668 (0.042)	0.563 (0.050)
GBLUP_IM_	0.475 (0.050)	0.677 (0.040)	0.566 (0.050)
GBLUP_H_	0.462 (0.052)	0.622 (0.045)	0.549 (0.051)
PGBLUP	0.582 (0.045)	0.727 (0.031)	0.623 (0.042)
PGBLUP_IM_	0.585 (0.044)	0.731 (0.031)	0.620 (0.043)
PGBLUP_H_	0.551 (0.047)	0.678 (0.036)	0.599 (0.044)
% Change relative to the P model			
RKHS	6.7	20.7	0.6
RKHS_IM_	5.2	20.6	−0.6
RKHS_H_	−2.6	11.9	−4.7
PRKHS	12.7	23.4	5.3
PRKHS_IM_	12.3	23.8	4.6
PRKHS_H_	5.7	15.6	1.6
GBLUP	−9.5	13.4	−4.3
GBLUP_IM_	−8.3	14.9	−3.8
GBLUP_H_	−10.8	5.6	−6.7
PGBLUP	12.4	23.4	5.9
PGBLUP_IM_	13.0	24.2	5.4
PGBLUP_H_	6.4	15.1	1.8

SE shown in parentheses. Average correlations are across 50 random partitions of the data with 70% in the training set and 30% in the validation set. Fitted models were pedigree (P), reproducing kernel Hilbert spaces regression without imputed GBS (RKHS), reproducing kernel Hilbert spaces regression with imputed GBS (RKHS_IM_), reproducing kernel Hilbert spaces regression with haplotype inference from imputed GBS (RKHS_H_), GBLUP without imputed GBS, GBLUPS_IM_ with imputed GBS, GBLUP_H_ with haplotype inferred from imputed GBS, pedigree reproducing kernel Hilbert spaces regression without imputed GBS (PRKHS), pedigree reproducing kernel Hilbert spaces regression with imputed GBS (PRKHS_IM_), pedigree reproducing kernel Hilbert spaces regression with haplotype inference from imputed GBS (PRKH_H_), pedigree GBLUP without imputed GBS (PGBLUP), pedigree GBLUPS_IM_ with imputed GBS (PGBLUPS_IM_), and pedigree GBLUP_H_ with haplotype inferred from imputed GBS (PGBLUP_H_). GBLUP, genomic best linear unbiased predictors; GBS, genotyping-by-sequencing.

In general, SEs of prediction for GY and ASI were approximately 0.04–0.05, whereas for AD they were approximately 0.03–0.04. Exceptions are the SEs of predictions for models fitted using inferred haplotypes, which are consistently high for all traits. Across imputation methods, the correlations of the models that used inferred haplotypes (H) gave the lowest prediction accuracy across methods, with GBS information, traits, and models (GBLUP_H_, PGBLUP_H_, RKHS_H_, PRKHS_H_) showing, as already mentioned, the largest SEs. Several correlations values are within the SE interval; therefore, it is adequate for describing patterns and trends rather than significances.

These results indicated, at least for this data set, the limited accuracy of using inferred haplotypes with GBS information. However, prediction correlation on imputed GBS based on 1000 flanking markers was similar to nonimputed GBS in several, but not all, cases. For example, PRKHS, PRKHS_IM_, PGBLUP, and PGBLUP_IM_ models yielded the best predictive correlations, with gains in predictive correlation relative to the pedigree (P) model of approximately 12% for GY and approximately 23% for AD. Models PGBLUP and PGBLUP_IM_ had the best predictive correlations for ASI, with gains relative to the P model of 5.9% and 5.4%, respectively (Table 3).

**Figure 2:**
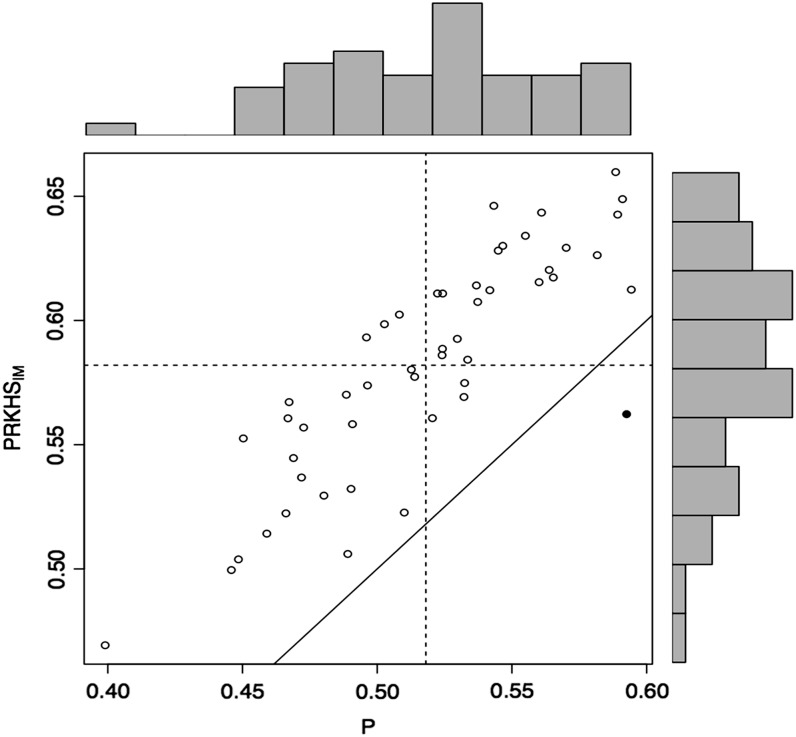
Plots of the predictive correlation for each of 50 cross-validation partitions for grain yield (GY) in experiment 1. When the best model is pedigree reproducing kernel Hilbert spaces regression with imputed genotyping-by-sequencing (PRKHS_IM_), this is represented by ○; when the best model is pedigree (P), this is represented by ●. The histograms depict the distribution of the correlations in the testing set obtained from 50 partitions for two models. The horizontal and vertical dashed lines represent the averages of the correlations for the testing sets in the 50 cross-validation partitions for the models shown in the Y axis and in the X axis, respectively. The solid line represents Y = X (when both models have the same prediction ability).

Figure 2 provides a scatter plot of the 50 correlations obtained with PRKHS_IM_ *vs.* those of the P model. A point above the 45-degree line implies that for that particular partition, the predictive correlation of the model on the vertical axis (PRKHS_IM_) is higher than that of the model on the horizontal axis (P). Although there is great variability in the correlations, the superior prediction accuracy of PRKHS_IM_ over the pedigree model is consistent across most partitions (*i.e.*, partitions that gave correlations of <0.5, those with correlations between 0.50 and 0.60, and those that produced correlations >0.6).

#### Prediction in experiment 2:

Estimates of broad-sense heritability and of prediction accuracy for this experiment are given in Table 4. As expected, estimates of broad-sense heritability and prediction accuracy were lower under drought stress conditions with a larger SE of the predictive correlation (0.088–0.107) than those obtained under WW (ample rainfall) conditions (0.057–0.071). In general, the correlation values are within the SE boundaries; therefore, we discuss prediction trends within the context of nonsignificant differences.

**Table 4:** Experiment 2 (296 maize lines), estimated broad-sense heritability and estimated average predictive ability between observed and predicted values for grain yield under drought stress and grain yield under well-watered conditions for 14 models

		Trait–Environment	
		Average Predictive Correlation of GY-SS	Average Predictive Correlation of GY-WW
Model			
RKHS		0.438 (0.093)	0.616 (0.059)
RKHS_IM_		0.442 (0.093)	0.616 (0.059)
RKHS_IMS_		0.454 (0.093)	0.606 (0.060)
RKHS_IML_		0.454 (0.093)	0.606 (0.060)
RKHS_H_		0.417 (0.087)	0.543 (0.060)
RKHS_HS_		0.449 (0.070)	0.595 (0.060)
RKHS_HL_		0.443 (0.094)	0.609 (0.057)
GBLUP		0.373 (0.103)	0.590 (0.071)
GBLUP_IM_		0.367 (0.107)	0.606 (0.070)
GBLUP_IMS_		0.385 (0.100)	0.612 (0.070)
GBLUP_IML_		0.385 (0.100)	0.612 (0.070)
GBLUP_H_		0.377 (0.088)	0.534 (0.064)
GBLUP_HS_		0.385 (0.100)	0.602 (0.070)
GBLUP_HL_		0.380 (0.100)	0.608 (0.065)
Estimated broad-sense heritability		0.380	0.441

SE shown in parentheses. Average correlations are across 50 random partitions of the data with 70% in the training set and 30% in the validation set. Fitted models were reproducing kernel Hilbert spaces regression without imputed GBS (RKHS), reproducing kernel Hilbert spaces regression with imputed GBS (RKHS_IM_), reproducing kernel Hilbert spaces regression with haplotype inference form imputed GBS (RKHS_H_), reproducing kernel Hilbert spaces regression with imputed GBS for short haplotypes (RKHS_IMS_), reproducing kernel Hilbert spaces regression with haplotype inference from imputed GBS with short haplotypes (RKHS_HS_), reproducing kernel Hilbert spaces regression with GBS imputation from long haplotypes (RKHS_IML_), reproducing kernel Hilbert spaces regression with haplotype inference from imputed GBS with long haplotypes (RKHS_HL_), GBLUP without imputed GBS, GBLUP with imputed GBS (GBLUP_IM_), GBLUP with haplotype inference from imputed GBS (GBLUP_H_), GBLUP with imputed GBS from short haplotypes (GBLUP_IMS_), GBLUP with haplotype inference from imputed GBS with short haplotypes (GBLUP_HS_), GBLUP with GBS imputation from long haplotypes (GBLUP_IML_), and GBLUP with haplotype inference from imputed GBS with long haplotypes (GBLUP_HL_). GBLUP, genomic best linear unbiased predictors; GBS, genotyping-by-sequencing; GY-SS, grain yield under drought stress conditions; GY-WW, grain yield under well-watered conditions.

For GY-SS, the prediction accuracy of GBLUP was consistently lower than that of RKHS models across all imputation methods, and it displayed larger SEs. Prediction differences within GBLUP and RKHS for the different imputation methods were very small. The best predictive models for GY-SS were for the imputed GBS models RKHS_IML_ and RKHS_IMS_. However, for GY under optimum conditions (GY-WW), prediction differences within GBLUPs and RKHSs for the different imputation methods were larger than for GY-SS. Models RKHS (with nonimputed GBS) and imputed GBS model RKHS_IM_ were the best predictors, closely followed by GBLUP_IMS_ and GBLUP_IML_.

**Figure 3:**
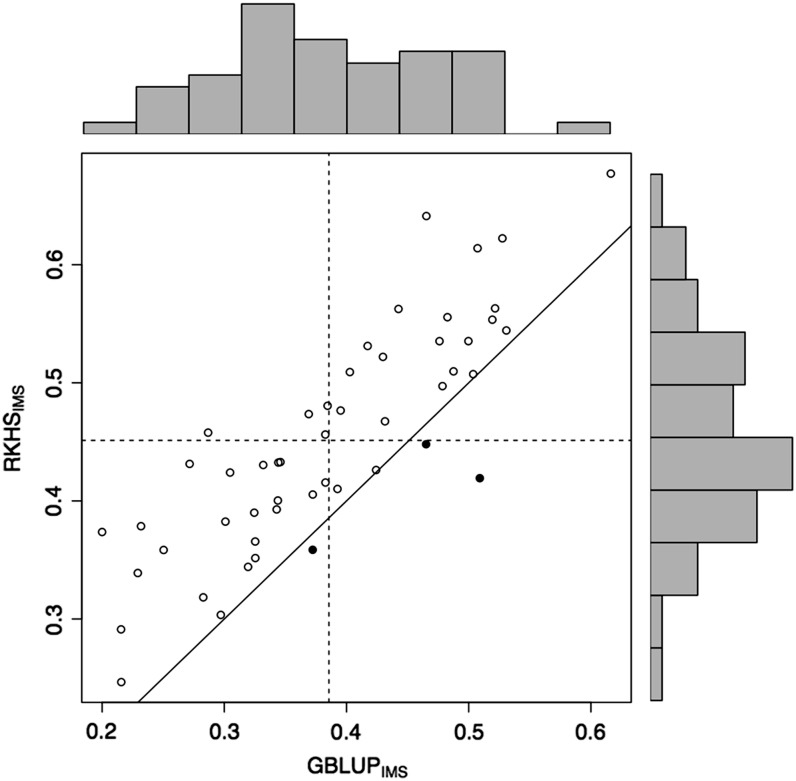
Plots of the predictive correlation for each of 50 cross-validation partitions for grain yield (GY) in the severe drought environment (SS; GY-SS) of experiment 2. When the best model is reproducing kernel Hilbert spaces regression with genotyping-by-sequencing (GBS) imputation from short haplotypes (RKHS_IMS_), this is represented by ○; when the best model is genomic best linear unbiased predictors with imputed GBS from short haplotypes (GBLUP_IMS_), this is represented by ●. The histograms depict the distribution of the correlations in the testing set obtained from the 50 cross-validation partitions for two models. The horizontal and vertical dashed lines represent the averages of the correlations for the testing sets in the 50 cross-validation partitions for the models shown in the Y axis and in the X axis, respectively. The solid line represents Y = X (when both models have the same prediction ability).

The variability of estimates of predictive correlation across data partitioned into training and testing sets was particularly high under severe stress (GY-SS), reflecting the lower precision obtained in field experiments under severe drought (also reflected in the lower broad-sense heritability and lower prediction accuracy). This dispersion of the actual differences in correlations between RKHS_IMS_ *vs.* GBLUP_IMS_ is shown in the scatter plots of the 50 random cross-validations in Figure 3. Most variability is attributable to the different partitions, and the prediction for RKHS_IMS_ surpassed those from GBLUP_IMS_ for most of the partitions. Results indicated that some random partitions picked subsets of training data that had lower correlations with observed values in the validation set (≤0.3), whereas other random partitions had closer relationships between the training and validation sets (≥0.5).

**Figure 4:**
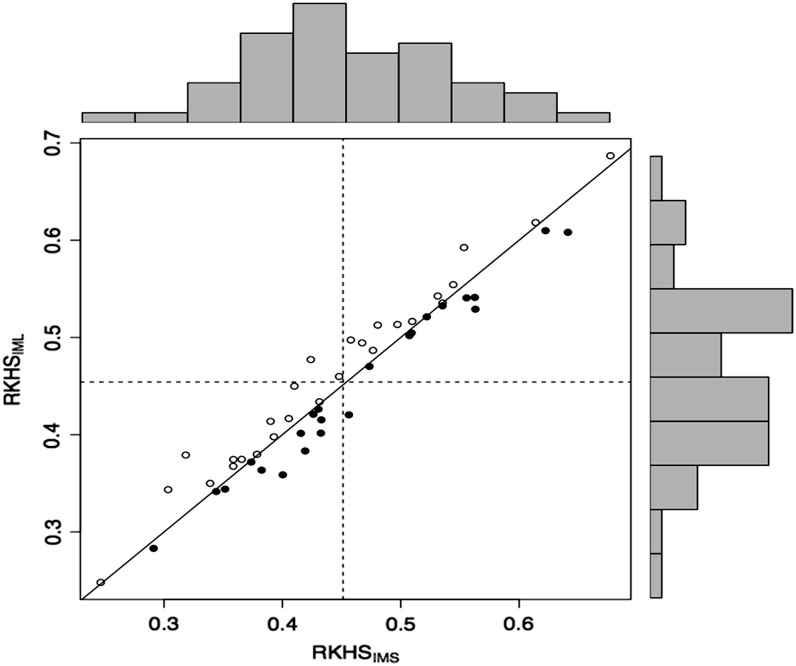
Plots of the predictive correlation for each of 50 cross-validation partitions for grain yield (GY) in the severe stress environment (SS; GY-SS) of experiment 2. When the best model is reproducing kernel Hilbert spaces regression with genotyping-by-sequencing (GBS) imputation from short haplotypes (RKHS_IMS_), this is represented by ●; when the best model is reproducing kernel Hilbert spaces regression with GBS imputation from long haplotypes (RKHS_IML_), this is represented by ○. The histograms depict the distribution of the correlations in the testing set obtained from the 50 cross-validation partitions for two models. The horizontal and vertical dashed lines represent the averages of the correlations for the testing sets in the 50 cross-validation partitions for the models shown in the Y axis and in the X axis, respectively. The solid line represents Y = X (when both models have the same prediction ability).

It is interesting to examine the dispersion among predictive correlations between short and long haplotype imputation methods for GY-SS (Figure 4 depicts RKHS_IMS_ *vs.* RKHS_IML_). As in Table 4, results from Figure 4 indicate that predictions of kernel models derived from 50 (short) and 100 (long) SNPs as flanking markers are very similar across all range of correlations (from <0.3 to almost 0.7). Furthermore, similar results indicate not much difference in prediction ability between short and long haplotype inference when using the GBLUP_IMS_ model for both GY-SS and GY-WW.

## Discussion

The overall aim of the study was to assess the prediction accuracy of GBS using two maize populations evaluated for different traits measured in different environments. Four specific questions were considered. How does the prediction accuracy of genomic models using GBS data compare with that of pedigree methods? Does the inclusion of pedigree information in the genomic model increase prediction accuracy? What is the impact of using imputation methods on the prediction accuracy attained by genomic models using GBS data? What is the prediction difference between the parametric GBLUP and the semi-parametric RKHS models?

Using GBS, one can obtain large numbers of markers at a relatively low cost and with potentially less ascertainment bias than that present in commercially available SNP arrays. There are concerns about using GBS markers in general regarding the cost of informatics, the error rate, and missing data issues. However, as modern SNP discovery pipelines such as Tassel, GBS Discovery Pipeline, SAMtools (Li *et al.* 2009), and SNVer (Wei *et al.* 2011) become available, many of the informatics requirements have been addressed for the users. Romay *et al.* (2013) compared GBS-SNP calls with Illumina array-based genotype values and estimated a mean discrepancy rate of 0.0118 between these two genotyping platforms. When analyzing maize inbred materials that are closely related to the B73 reference genome, such as our DH lines, excluding heterozygote calls further reduces the discrepancy rate to 0.0058, suggesting the reliability of GBS when reference genome information is available. However, interpreting missing data from GBS markers attributable to the low coverage might still be challenging for some applications, especially genome-wide association analysis and GS. Further, incorporating GBS data in genomic prediction is not free of hurdles and, to our knowledge, no empirical evaluation has yet been performed regarding the use of GBS genotypes for genomic-enabled prediction.

### Dealing with missing genotypes in GBS data

Typically, GBS data contain high proportions of missing genotypes, which poses a real challenge. In this study, we proposed and evaluated two approaches to face that challenge. First, we proposed models based on called genotypes only circumventing imputation. Second, we developed and implemented several imputation methods for GBS data and evaluated the performance of prediction approaches using imputed data. Phasing was greatly facilitated by the fact that the data were from pure lines, given that the imputation of genotypes with large percentages of heterozygous loci is considerably more challenging.

Results from experiment 1 indicated, for the three traits, a very slight increase in prediction (not significant) of models using imputed GBS as compared to those with uncalled GBS, but not for models with inferred haplotypes. Results from experiment 2 indicated no prediction improvement on the imputed GBS. The average gains in prediction accuracy varied greatly across traits and imputation methods, and in some cases methods that use imputed genotypes or inferred haplotypes were outperformed by methods based on called genotypes only. Clearly, if GBS is to become a common genotyping approach in breeding programs, then further research of developing and calibrating imputation methods for GBS data are needed.

### Prediction accuracy using GBS data

Successful prediction accuracies in models using GBS data in wheat were recently reported (Poland *et al.* 2012). In this study, we provide the first extensive evaluation of genomic-enabled prediction using GBS on real maize phenotypic data. We considered two maize data sets: three traits were evaluated in experiment 1 and one trait was evaluated under three environmental conditions in experiment 2. Importantly, these two data sets have very different genetic structures and, therefore, represent very different prediction problems. Experiment 1 comprises full-sib and other related families, whereas experiment 2 includes a diverse panel of lines from the global maize breeding program of CIMMYT, some of which are distantly related.

### Prediction accuracy with family data

In experiment 1, results show that combining GBS with pedigree data yields consistent gains in prediction accuracy, relative to pedigree-based predictions. These gains ranged from modest (2–5%) to substantial (10–24%), depending on the trait (regardless of whether GBS genotypes were imputed) and on the statistical method used to incorporate GBS data (either GBLUP or RKHS). Our results are in agreement with previous reports of consistent gains in prediction accuracy when pedigree was combined with genomic information derived from hybridization-based genotyping methods in maize and wheat (*e.g.*, de los Campos *et al.* 2009; Crossa *et al.* 2010; Burgueño *et al.* 2012; Heslot *et al.* 2012). The fact that combining GBS and pedigree data leads to higher prediction accuracy than using pedigree data alone indicates that GBS data capture information beyond what can be captured by the pedigree only, which in the case of experiment 1 reduces to the mean of the family. Unlike pedigree information, the use of marker data allows capturing within-family differences and, to some extent, bridging the signal of genetic similarity detected by genetic markers, which might have been overlooked by pedigree information.

It should be mentioned that prediction from the pedigree GBLUP model operates not only within family means but also between families by capturing family structure. Furthermore, GBLUP and RKHS captured population structure and substructures (because of different parental relationships), as well as within and between family means, because regressing the phenotypic values on all marker values is equivalent to regressing phenotypes on all marker-derived principal components (de los Campos *et al.* 2010; Janss *et al.* 2012).

Models using GBS data with no pedigree information were systematically outperformed by those combining pedigree and GBS data. Similar results were reported before using low-density markers in wheat data (de los Campos *et al.* 2009; Crossa *et al.* 2010). However, other studies have shown that as marker density increases, the benefits of combining pedigree and genotype information diminish, relative to models using marker information only, until a point is reached at which, above a given marker density, combining pedigree and markers yields a prediction accuracy similar to the one obtained with markers only (Vazquez *et al.* 2010).

Populations with extensive LD are also expected to gain high prediction accuracy because of the linkage between tagged SNPs with the unobserved casual genetic polymorphism (or QTL) (Zhong *et al.* 2009; Brito *et al.* 2011). The maize genome reportedly suggests rapid LD decay in both tropical and temperate germplasm (Lu *et al.* 2011; Romay *et al.* 2013), indicating a higher number of genetic markers are needed to arrive at the same prediction power. When applying genomic prediction models to closely related breeding materials (such as the families in experiment 1), in theory, considerable prediction accuracy can be expected because family linkages, low levels of diversity, small number of meiosis, and relatively large LD occurred in the genome.

### Prediction accuracy in a diverse panel

Experiment 2 comprises a collection of maize lines with no close family relationships. However, these lines have different degrees of genetic similarity that could be described as a combination of population structure, substructure, and cryptic relationships, all of which are captured by markers and consequently accounted for in genomic regressions such as GBLUP (Janss *et al.*, 2012) or RKHS (de los Campos *et al.* 2010).

In this experiment, pedigree information is not available; therefore, comparing marker *vs.* pedigree prediction is not possible. However, our results can be compared with those of others who used this data set for genomic-enabled prediction using SNP arrays (*e.g.*, Crossa *et al.* 2010; González-Camacho *et al.* 2012; Windhausen *et al.* 2012). In our study, prediction accuracy using GBS data for GY under WW conditions ranged from 0.544 to 0.624, depending on the model and depending on whether GBS data were imputed. Windhausen *et al.* (2012) used 55,000 SNPs and experiment 2 data with the GBLUP model, and achieved a prediction accuracy of 0.50 for GY in optimum environments. Crossa *et al.* (2010), using experiment 2 with only 1148 SNPs, reported predictions of approximately 0.51 for GY-WW for RKHS, Bayesian-LASSO, and GBLUP; however, González-Camacho *et al.* (2012), using experiment 2 data with 55,000 SNPs, obtained predictions of approximately 0.55 for GY-WW using linear and nonlinear models. Regardless of the prediction model, GBS information seemingly performs better than other genotyping platforms with or without imputation for traits under WW conditions.

In experiment 2, the prediction accuracy of GY under stressed conditions ranged from 0.373 to 0.4541, depending on whether GBS data were imputed and depending on the statistical model used. These correlations are considerably lower than those obtained under WW conditions and are consistent with previously reported results for this trait under these conditions. For instance, using this same data set but only 1,148 SNPs, Crossa *et al.* (2010) found prediction accuracies ranging from 0.42 to 0.45 for GY-SS.

Part of the differences across studies may be attributable to the specifics of the validation designs used; however, the similarities of results across traits, environmental conditions, and studies allow us to conclude that, at a minimum, prediction accuracy with GBS-imputed data are on par with that of 55,000 SNPs, with the advantage that GBS data are less expensive.

### Parametric *vs.* semi-parametric methods

In our study, we compared two ways of incorporating GBS data into models: parametric method (GBLUP) and semi-parametric method (RKHS). In general, no single method appeared to be clearly superior. Overall, in models using GBS data only (*i.e.*, without pedigree information), RKHS performed slightly better than GBLUP, with the only exception of GY under WW conditions in experiment 2. However, when GBS data were combined with pedigree information, prediction accuracy increased and the difference between the methods (GBLUP *vs.* RKHS) diminished. Overall, our results are in agreement with those of other authors who reported only small differences in the predictive performance of different statistical methods (Heslot *et al.* 2012). However, still other authors (Crossa *et al.* 2010; González-Camacho *et al.* 2012) have found differences across methods depending on the genetic architecture of the trait and depending on marker density. In many previously published studies, low marker density was pointed out as a possible reason for the lack of differences across methods; essentially, poor LD between markers and QTL at low marker density acts as a factor limiting overall prediction accuracy and the ability of each method to express its potential. Here, we have much higher density; however, because of the large proportion of imputed genotypes, rapid LD decay and weak LD between GBS-derived markers and causal loci may limit overall prediction accuracy and the ability for each of the models to show differences.

Including all pairwise (or higher-order) interactions among markers in linear models continues to be a difficult problem because there are thousands of cryptic gene × gene interactions with very small effects. Although parametric linear regression imposes a linear relationship between markers and phenotype, nonparametric models do not impose any assumptions on the phenotype–genotype relationship, therefore making it possible to capture interactions among loci. The results of this study using GBS in two maize data sets show that linear regression and nonparametric models provided similar prediction correlations for most trait–environment combinations, except for GY under drought conditions (GY-SS), in which GBLUP predictions were lower than those from RKHS. However, this trend changed under WW conditions (GY-WW), in which both models performed very similarly in terms of prediction accuracy.

## Conclusions

Our results indicate that prediction of the performance of inbred maize lines using GBS data combined with pedigree information outperforms the prediction accuracy of pedigree-based methods and, at a minimum, can yield prediction accuracies comparable to those obtained with low-density to mid-density SNP arrays. The relatively low cost of GBS data further makes GBS a competitive alternative for improving the efficiency of breeding programs. Regarding the models, we found no clear differences in the prediction accuracy of the two models; however, even though RKHS was slightly better than GBLUP when using GBS alone, both models performed similarly with pedigree information. Dealing with missing genotypes poses an important challenge when using GBS data, and in this study we propose several ways of confronting that challenge. Results of this study indicated that prediction based on imputed SNPs and/or inferred haplotypes does not significantly improve predictions based on nonimputed SNPs. Further research is needed to develop and calibrate effective imputation methods for GBS data and to compare their predictive ability with those of models that use only uncalled GBS.

## Appendix A

### Statistical models

The methods used in this study (including pedigree methods), GBLUP, and RKHS can all be cast within a general class of Gaussian random effects models. The general form of these models can be described as follows:

[1] $$\{\begin{array}{c} y_{i}=g_{i}+\varepsilon_{i}\\ \varepsilon_{i}\overset{iid}{∼}N(0,\sigma_{\varepsilon }^{2}), \end{array}$$

where $y_{i}$ is the phenotype of the *i^th^* individual (*i=*1,…*n)*, $g_{i}$ is a genetic value, and *ε_i_* represents model residuals, all following the same normal distribution. For ease of notation, we have ignored the intercept and other nongenetic effects. Extensions used to accommodate nongenetic effects are straightforward.

In the model described in equation [1], the separation of signal $\left(g_{i}\right)$ from noise $\left(\varepsilon_{i}\right)$ is achieved by assigning different distributions to the vector of model residuals and the vector of genetic values. All the models used in this study share the sampling model described by equation [1]. In vector notation, we have

[2] $$p(y|g,\sigma_{\varepsilon }^{2})=N(y|g,I\sigma_{\varepsilon }^{2}),$$

where $y=\{y_{i}\}\;$and $\;g=\{g_{i}\}$ are vectors of phenotypes and genetic values, respectively. However, the different models differ regarding the prior probability distributions assigned to genetic values. In its most general formulation, and following the study by de los Campos *et al.* (2010), the vector of genetic values can be represented as the sum of $N_{k}$ random effects as follows

[3] $$g=\sum_{k=1}^{N_{k}}g_{k},$$

where each of these random effects follow independent normal distributions of the form

[4] $$g_{k}∼N(0,\sigma_{g_{k}}^{2}K_{k}),$$

where $K_{k}$ is the *n*×*n* covariance matrix computed either from markers or from pedigree. Combining expressions [2] through [4], we find that the joint distribution of the phenotypes and of the random effects is given by

[5] $$p(y,g_{1},\ldots ,g_{N_{k}}|\sigma_{\varepsilon }^{2},\sigma_{g_{1}}^{2},..,\sigma_{g_{N_{k}}}^{2})=N\left(y|\sum_{k=1}^{N_{k}}g_{k},I\sigma_{\varepsilon }^{2}\right)\prod_{k=1}^{N_{k}}N(g_{k}|0,K_{k}\sigma_{g_{k}}^{2}),$$

The fully Bayesian model is completed by assigning a prior density for variance parameters. Following the standard assumption, we assigned scaled-inverse chi-squared densities to each of the variance parameters

[6] $$\begin{array}{l} p\left(y,g_{1},\ldots ,g_{N_{k}},\sigma_{\varepsilon }^{2},\sigma_{g_{1}}^{2},..,\sigma_{g_{N_{k}}}^{2}|H\right)=N\left(y|\sum_{k=1}^{N_{k}}g_{k},I\sigma_{\varepsilon }^{2}\right)\chi^{-2}\left(\sigma_{\varepsilon }^{2}|df_{\varepsilon },S_{\varepsilon }\right)\\ \;\times \prod_{k=1}^{N_{k}}N\left(g_{k}|0,K_{k}\sigma_{g_{k}}^{2}\right)\chi^{-2}(\sigma_{g_{k}}^{2}|df_{k},S_{k}), \end{array}$$

where *H* denotes all hyperparameters indexing the prior probability distributions. The models used in this study are all special cases of the model described in equation [6]. Table A2 lists all models used, the number of random effects included in each model, and the covariance matrices used.

**Table A2:** Models, number of random effects for each model, and covariance matrices associated with the random effects

Model	Number of Random Effects $\left(N_{k}\right)$	Covariance Matrices
Pedigree	1	**A**
GBLUP	1	**G**
PGBLP	2	**A, G**
RKHS	3	**K_1_, K_2_, K_3_**
PRKHS	4	**K_1_, K_2_, K_3_, A**

**A,** numerator relationship matrix derived from a pedigree; **G,** genomic relationship matrix derived from either imputed markers using cross-products of marker genotypes; **K_k_** (*k* = 1, 2, 3), Gaussian kernel computed from marker genotypes.

### Pedigree model

In the pedigree model, the matrix **A** is a numerator relationship matrix (twice the matrix of kinship coefficients) computed from a pedigree.

### GBLUP

In standard GBLUP (VanRaden 2007, 2008), the entries of the genomic relationship matrix, Gii′, represent proportions of allele sharing between pairs of individuals (*ii′*), realized at markers. Commonly, these are computed using cross-products of marker genotypes of the form

[7] G=ii′∑j=1p(xij−qj)(xi′j−qj)∑j=1pqj(1−qj),

where $x_{ij}\in \{0,1\}$ represents the marker covariates (*i=*1,…,*n* individuals and *j=*1,…,*p* markers) and $q_{j}$ is the estimated allele frequency computed from nonmissing genotypes. Let $z_{ij}=x_{ij}-q_{j},\;i=1,\ldots ,n;j=1,\ldots ,p$; then, equation [7] in matrix notation can be expressed as $G=ZZ\prime /\sum_{j=1}^{p}q_{j}\left(1-q_{j}\right)$. This formulation assumes that all genotypes are available. With GBS data, there is a large proportion of marker genotypes that are missing. To circumvent this problem we considered imputation, but even after imputation uncalled genotypes were still present, so the missing genotypes were replaced by their expected values and the proportion of missing genotypes for each individual was taken into account using a modified version of equation [7] given by VanRaden (2008):

[8] $$G=\frac{WZZ\prime W}{\sum_{j=1}^{p}q_{j}(1-q_{j})},$$

where **W** is a diagonal matrix whose entries are given by $w_{ii}=\sqrt{\frac{\sum_{j=1}^{p}q_{j}(1-q_{j})}{\sum_{j\in S_{i}}q_{j}(1-q_{j})}}$where $S_{i}$denotes the set of markers for which genotypes were observed at individual *i*.

### Reproducing kernel Hilbert spaces models with kernel averaging

In this implementation, we used three random effects. The covariance structures used were computed by means of a Gaussian kernel; therefore, the (*ii′)* of the kernels were given by K=k,ii′exp{−hkdii′2}, where $h_{k}$ is a bandwidth parameter peculiar to the *k^th^* reproducing kernel and $d_{ii\prime }^{2}$ is the genetic distance between lines *i* and *i′* computed as follows: $d_{ii\prime }^{2}=\sum_{j=1}^{p}{\left(x_{ij}-x_{i\prime j}\right)}^{2}$. The bandwidth parameters were chosen to have an extremely local kernel (*i.e.*, one in which the covariance function declines very fast as genetic distance increases), one intermediate kernel, and one kernel in which the covariance function gives a high degree of resemblance even between distantly related lines. Specifically, we chose h1=5/q0.05,   h2=1/q0.05,  h3=15q0.05, where is the fifth percentile of $d_{ii\prime }^{2}$.

As with GBLUP, the computation of the Gaussian kernel requires observed marker genotypes at pairs of lines. With GBS, several marker genotypes are missing and, even after imputation, uncalled genotypes are still present. To cope with missing genotypes, the computation of distances was based only on observed genotypes, that is: ${\tilde{d}}_{ii\prime }^{2}=\sum_{j\in S_{ii^{\prime }}}^{}{\left(x_{ij}-x_{i\prime j}\right)}^{2}/p_{ii\prime }$ where $p_{ii\prime }$ represents the number of observed genotypes for lines *i* and *i′*.

### Implementation

Samples from the posterior distribution, equation [6], were obtained using the Gibbs sampler described by de los Campos *et al.* (2010). The software was implemented in R (R Core Team 2013) and is available on request. The degree-of-freedom parameters of the scaled inverted chi-squared distributions were 5, and the scale parameters were chosen to match a heritability of 0.5, that is: $S_{\varepsilon }=0.5\times \left(df-2\right)V_{y}$ and $S_{k}=\frac{0.5\times \left(df-2\right)V_{y}}{N_{k}},$ where $V_{y}$ is the sample variance of $\left\{y_{i},i=1,\ldots ,n\right\}$. Posterior means were estimated using 30,000 samples obtained after discarding 5000 that were taken as burn-in.
